# Case Report: A reversible autonomic–endothelial migraine pattern: a conceptual perspective illustrated by a clinical case

**DOI:** 10.3389/fpain.2026.1748356

**Published:** 2026-04-14

**Authors:** Vlodeks Gromakovskis

**Affiliations:** 1 Pain Medicine Unit, Neurology Department, Association of Health Centers (Veselības Centru Apvienība, VCA), Riga, Latvia; 2 Independent Pain Medicine Researcher, Riga, Latvia

**Keywords:** antihypertensive therapy, autonomic imbalance, endothelial dysfunction, migraine, neurovascular coupling, sneezing, vasomotor rhinitis

## Abstract

Migraine is commonly regarded as a primary neurovascular disorder; however, systemic endothelial and autonomic factors may modulate its expression and course. A 41-year-old male with a 10-year history of migraine without aura and vasomotor rhinitis experienced sustained remission after initiation of nebivolol 5 mg and (perindopril 5 mg/indapamide 1.25 mg/amlodipine 5 mg) once daily for mild hypertension. Attacks occurred 3–4 times per month—often on Saturdays—were triggered by strong odors such as perfume, and occasionally accompanied by nausea or vomiting. Headaches fulfilled ICHD-3 criteria for migraine without aura and initially responded to triptans. Within two weeks of therapy, blood pressure normalized and both migraine and nasal symptoms resolved. Remission persisted for 12 months. The temporal sequence and clinical pattern appear consistent with a clinical improvement that may involve endothelial and autonomic modulation, among several plausible contributors, including combined antihypertensive therapy. The patient's blood pressure remained within the physiological range throughout the observation period, indicating that the sustained remission extended beyond antihypertensive control *per se*. This case illustrates a hypothesis-generating clinical pattern potentially involving endothelial and autonomic modulation and highlights how vascular homeostasis and circadian autonomic factors may influence migraine chronification and remission.

## Graphical Abstract

**Figure:**
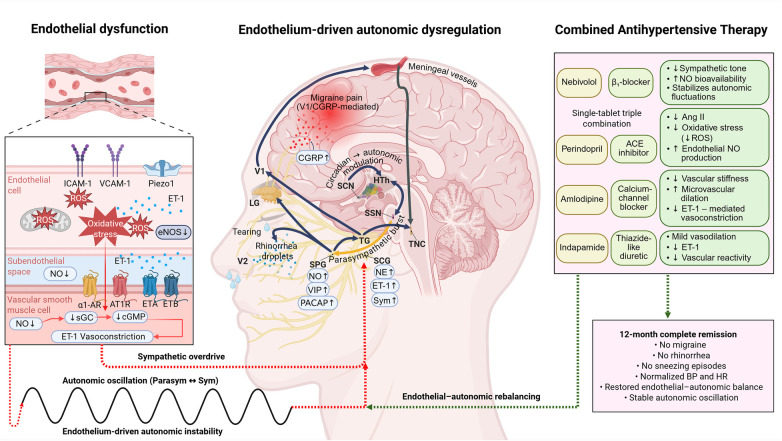
Conceptual diagram illustrating the hypothesized endothelial–autonomic interplay contributing to the observed migraine pattern in this clinical case. This schematic integrates systemic endothelial dysfunction with downstream effects on autonomic regulation, trigeminovascular pathways, and clinical expression of migraine in this case. The left panel depicts endothelial dysfunction characterized by reduced nitric oxide (NO) bioavailability, elevated endothelin-1 (ET-1), oxidative stress (ROS↑), adhesion molecule expression (ICAM-1/VCAM-1), and impaired eNOS signaling—changes commonly associated with hypertension, psychological stress, sympathetic overactivity, and systemic inflammation. These endothelial perturbations contribute to autonomic instability (parasympathetic–sympathetic oscillation) and sympathetic overdrive (SCG-mediated NE↑, ET-1↑). The central panel illustrates how autonomic dysregulation interfaces with trigeminal (TG–V1) and sphenopalatine ganglion (SPG) pathways. Parasympathetic bursts from the superior salivatory nucleus (SSN) drive tearing, rhinorrhea, and sneezing episodes, while convergent autonomic and trigeminal influences facilitate CGRP-mediated activation of dural meningeal vessels, leading to V1-distributed migraine pain. Circadian–autonomic interactions (SCN → hypothalamus) and sympathetic surges further modulate this network. The right panel summarizes the patient's treatment with combined antihypertensive therapy (nebivolol, perindopril, amlodipine, indapamide), which is shown conceptually as restoring endothelial–autonomic balance (improved NO, reduced ET-1/ROS, stabilized autonomic oscillation). This therapeutic normalization aligns with the observed 12-month complete remission, including resolution of migraine, rhinorrhea, sneezing episodes, and normalization of blood pressure and heart rate. *The figure is intended to aid conceptual understanding and does not imply causal or mechanistic certainty. Temporal relationships illustrated in the schematic are conceptual and do not represent real-time physiological sequencing.* ACC, anterior cingulate cortex; ACEI, angiotensin-converting enzyme inhibitor; ANG II, angiotensin II; ASICs, acid-sensing ion channels; α-SMA, alpha-smooth muscle actin; α1-AR, alpha-1 adrenergic receptor; AT1R, angiotensin II type-1 receptor; BP, blood pressure; cGMP, cyclic guanosine monophosphate; CGRP, calcitonin gene-related peptide; eNOS, endothelial nitric oxide synthase; ET-1, endothelin-1; ETA/ETB, endothelin A/B receptors; HTh, hypothalamus; HR, heart rate; ICAM-1, intercellular adhesion molecule-1; LG, lacrimal gland; NE, norepinephrine; NO, nitric oxide; PACAP, pituitary adenylate cyclase-activating peptide; Parasym, parasympathetic activity; Piezo1, mechanosensitive ion channel; ROS, reactive oxygen species; SCG, superior cervical ganglion; SCN, suprachiasmatic nucleus; sGC, soluble guanylate cyclase; SPG, sphenopalatine ganglion; SSN, superior salivatory nucleus; Sym↑, increased sympathetic activity; TG, trigeminal ganglion; TNC, trigeminal nucleus caudalis; V1/V2, ophthalmic and maxillary divisions of the trigeminal nerve; VCAM-1, vascular cell adhesion molecule-1; VIP, vasoactive intestinal peptide.

## 1 Introduction

Migraine is a complex neurovascular disorder involving activation of the trigeminovascular system and release of calcitonin gene-related peptide (CGRP), nitric oxide (NO), and other vasoactive mediators (1, 2). Current concepts increasingly recognize systemic vascular and autonomic factors as potential contributors to attack generation and chronification (3–6). Endothelial dysfunction may lead to abnormal NO bioavailability, oxidative stress, and altered microvascular reactivity, creating a permissive milieu for trigeminovascular activation (7–10).

Autonomic imbalance—typically sympathetic overactivity and reduced parasympathetic tone—has been linked to vascular instability and enhanced CGRP release (11, 12). These mechanisms help explain why agents that restore vascular and autonomic balance, such as β-blockers, ACE inhibitors, and calcium-channel blockers, may prevent migraine even without substantial blood-pressure changes (13–18).

This report describes an episodic migraine presentation with prominent cranial-autonomic manifestations and sustained remission following combined antihypertensive therapy, without implying causal attribution.

### 1.1 Case description

This case report was prepared in accordance with the CARE (CAse REport) guidelines (19). A 41-year-old male had experienced pulsatile, moderate-to-severe headaches for approximately 10 years. The attacks fulfilled ICHD-3 criteria for migraine without aura. Episodes occurred about 3 to 4 times per month, most often on Saturdays, suggesting a possible behavioural-circadian trigger pattern. Each attack lasted 6–24 h and was accompanied by photophobia, phonophobia, and bilateral nasal congestion with watery rhinorrhea.

Strong odors such as perfume frequently precipitated attacks, and some episodes were accompanied by nausea and occasional vomiting. During many attacks the patient sneezed repeatedly—sometimes several times in succession - and both eyes watered profusely, indicating bilateral cranial autonomic activation. Outside of acute headache attacks, the patient also reported occasional episodes of spontaneous cranial-autonomic activity, including sneezing and rhinorrhea sometimes accompanied by mild unilateral head discomfort on awakening. In retrospect, mild dull head discomfort sometimes appeared during the night or very early in the morning, preceding the patient's awakening, whereas nasal congestion was typically noticed only upon awakening. Typically, these episodes began abruptly with multiple repetitive sneezes often 7–10 in succession—followed by profuse watery nasal discharge that could persist for hours until sleep, despite no response to antihistamines or decongestants. The attacks appeared spontaneously, without obvious allergic or environmental triggers. They represented an intense parasympathetic outflow episode culminating in nasal congestion and marked mucosal edema. These episodes typically resolved after rest or sleep, but in some instances a migraine attack occurred 24–48 h later, when the rhinorrhea had ceased and nasal breathing normalized. Such episodes did not always precede or follow headaches, suggesting a dynamic autonomic fluctuation rather than a fixed prodrome. The pattern was interpreted as transient parasympathetic hyperactivity followed by delayed sympathetic rebound, which could lower the migraine threshold. These features, together with triptan responsiveness, were consistent with a migraine rather than a hypertensive phenotype.

Ibuprofen 400–600 mg initially provided partial relief, but efficacy declined over time. Sumatriptan 50–100 mg remained effective for several years, but during the later phase of treatment it was consistently followed, about 30–40 min after intake, by a transient episode of autonomic arousal and sensory hypersensitivity. During this period the patient reported diffuse muscle aching and twisting-like discomfort, as well as mild pain to normally non-painful stimuli—such as washing the face or swallowing water or tea—unrelated to temperature. These sensations gradually subsided over 2 to 4 h and never occurred spontaneously in the absence of triptan use.

At age 41, routine evaluation revealed persistent mild arterial hypertension (≈150/95 mm Hg) and resting tachycardia (>90 bpm). The patient also reported increasing fatigue and more frequent headaches. Combined antihypertensive therapy was started: The patient received nebivolol 5 mg (Nebilet®, Berlin-Chemie/Menarini, Berlin, Germany; Florence, Italy) and a fixed-dose combination of perindopril arginine 5 mg, indapamide 1.25 mg, and amlodipine 5 mg (Triplixam®, Servier, France) once daily. No other prophylactic drugs (anticonvulsants, antidepressants, botulinum toxin, or CGRP monoclonal antibodies) were used before or during follow-up.

Within two weeks of therapy initiation, blood pressure stabilized (≈130/85 mm Hg), resting heart rate decreased to ≈74 bpm, and both headache and nasal/tearing symptoms resolved. Before therapy, attacks and nasal swelling had often abated after sleep, suggesting a sleep-mediated autonomic reset; after treatment this fluctuation disappeared. Hemodynamic and symptomatic changes during follow-up are shown in Table 1.

**Table 1:** Dynamic changes in blood pressure, heart rate, and symptoms during 12 months of follow-up.

Parameter	Baseline	2 weeks	1 months	3 months	6 months	12 months
Blood pressure (mm Hg)	150/95	130/85	125/80	125/80	125/80	125/80
Heart rate (bpm)	92	74	70	70	70	70
Migraine attacks/month	3–4	1	0	0	0	0
Rhinorrhea/nasal congestion	Daily	Occasional	Absent	Absent	Absent	Absent
Sneezing/lacrimation episodes	Frequent	Rare	Absent	Absent	Absent	Absent

The patient maintained the same regimen for 12 months with stable blood pressure (BP) (≈125/80 mm Hg) and heart rate (HR) 68–72 bpm. No migraine, rhinorrhea, or autonomic symptoms recurred. Daily home BP and HR measurements were recorded using a validated monitor, and attack frequency was logged in a prospective diary. No adverse events were reported. At follow-up visits (every three months), clinical and hemodynamic parameters were re-evaluated and remained stable, with no return of nasal or headache symptoms.

Reports of complete and durable remission under combined antihypertensive therapy are rare but noteworthy, as they offer insight into potential secondary endothelium-dependent clinical pattern within the migraine spectrum (20–24). Key distinguishing features between typical primary migraine and the endothelium-modulated pattern observed in this case are summarized in Table 2.

**Table 2:** Primary migraine vs endothelium-modulated migraine pattern.

Feature	Typical Primary Migraine	Endothelium/Autonomic-Modulated Pattern
Onset	Adolescence/early adulthood	Mid-life (≈40 years)
Frequency	1–2/month	3–4/month (weekend-predominant)
Triggers	Stress, hormonal change	Odors, sleep shift, vascular factors
Autonomic signs	Minimal	Rhinorrhea, tearing, sneezing
Triptan response	Stable	Early good, later partial
Blood pressure	Normal	Mildly elevated
Preventive response	Variable	Remission with antihypertensives

The present case describes a middle-aged man with long-standing migraine and vasomotor rhinitis who achieved 12-month remission after initiation of nebivolol and perindopril arginine + indapamide + amlodipine. The case may illustrate a reversible interaction between endothelial and autonomic systems that modulate migraine expression.

## 2 Discussion

This report illustrates a reversible migraine pattern in which the observed clinical course is consistent with systemic endothelial and autonomic modulation, without implying mechanistic primacy or causality. Complete cessation of attacks under combined antihypertensive therapy is consistent with the possibility that modulation of vascular and autonomic homeostasis may influence migraine expression. Although endothelial and autonomic dysfunction have been implicated in migraine pathophysiology, documented cases linking both mechanisms with full remission remain rare. This case emphasizes the importance of considering systemic endothelial and autonomic homeostasis when evaluating refractory or atypical migraine presentations. It bridges clinical observation with experimental evidence of neurovascular and autonomic coupling, offering a translational perspective on migraine as a disorder of systemic regulation.

### 2.1 Differentiation from hypertensive headache

Although mild hypertension was present, the headache characteristics—pulsatile quality, photophobia, phonophobia, osmophobia, occasional nausea or vomiting, and triptan responsiveness—fulfilled ICHD-3 criteria for migraine without aura (25, 26). These features differ markedly from the dull, pressure-like pain seen during acute blood-pressure spikes (25). Attacks had occurred for years before hypertension was detected, further supporting a primary migraine presentation with secondary vascular modulation. The comparative clinical features distinguishing migraine from hypertensive headache are summarized in Table 3.

**Table 3:** Migraine vs hypertensive headache.

Criterion	Migraine	Hypertensive Headache
Pain type	Pulsatile, moderate–severe	Dull, diffuse, pressure-like
Duration	4–72 h	Variable, often continuous
Associated symptoms	Photophobia, nausea, vomiting, osmophobia	None
Autonomic features	Possible	Absent
Triggers	Odors, sleep change	Acute BP rise
Triptan response	Yes	No
Temporal relation to BP	Precedes HTN onset	Coincides with crisis

BP, blood pressure; HTN, hypertension; h, hours.

### 2.2 Endothelial and autonomic modulation

Nebivolol enhances nitric-oxide bioavailability and attenuates sympathetic overdrive; perindopril, amlodipine, and indapamide improve endothelial reactivity and decrease oxidative stress (27–31). The primary pharmacological mechanisms and their endothelial–autonomic relevance are summarized in Graphical Abstract. Together, these effects may be associated with normalization of vascular tone, reduced CGRP signaling, and stabilization of neurovascular coupling (9, 32, 33). This endothelial–autonomic modulation may represent one of several plausible contributors to the rapid remission observed, extending beyond mere blood-pressure normalization. Transient sensory hyperresponsiveness could reflect a short-lasting central sensitization triggered by serotonergic vascular modulation (34).

Although the hypertension was mild, the combination of perindopril arginine + indapamide + amlodipine and nebivolol was selected to achieve gentle blood-pressure control together with autonomic stabilization. According to current ESC/ESH guidelines, such combinations are acceptable in patients with elevated heart rate, sympathetic predominance, or metabolic risk. In this case, the treatment choice was driven by both cardiovascular and autonomic considerations, resulting in optimal tolerance and stable hemodynamic parameters.

Importantly, the simultaneous initiation of multiple antihypertensive agents represents a major confounding factor. The relative contribution of individual drugs cannot be disentangled, and the observed remission may reflect combined central, peripheral, and nonspecific preventive effects rather than endothelial modulation *per se*. Importantly, nebivolol itself represents an evidence-based antimigraine prophylactic agent; therefore, the observed remission cannot be attributed to endothelial–autonomic mechanisms alone and likely reflects overlapping pharmacological effects.

### 2.3 Endothelial–neurovascular continuum

The migraine mechanism may involve a bidirectional “endothelial–neurovascular continuum,” wherein endothelial cells and perivascular trigeminal afferents communicate through NO, CGRP, endothelin-1, and inflammatory mediators (35, 36). Endothelial stress may lower the threshold for trigeminovascular activation; conversely, improved endothelial function may restore inhibitory control and raise the migraine threshold. This model aligns with current experimental data on vascular–neuronal cross-talk (37, 38).

### 2.4 Circadian endothelial resetting

The patient's symptoms often subsided after sleep, and attacks clustered on weekends. These patterns are consistent with a possible *circadian endothelial resetting* phenomenon—night-time parasympathetic dominance and reduced sympathetic output may restore vascular tone and limit CGRP release (39, 40). Sleep irregularities or behavioural changes on weekends could destabilize this rhythm and precipitate attacks, consistent with recent evidence on circadian modulation of migraine susceptibility (41, 42).

Clinicians should note that recurrent post-sleep resolution of migraine and nasal symptoms may signal a circadian autonomic mechanism responsive to endothelial modulation (43, 44).

### 2.5 Autonomic oscillation and interictal cranial symptoms

Beyond circadian patterns, the patient exhibited intermittent cranial-autonomic episodes—rhinorrhea and sneezing—that occurred independently of pain and, in some instances, 24–48 h before migraine onset. Such interictal phenomena may reflect a low-frequency fluctuation of trigemino-parasympathetic activity rather than a fixed migraine prodrome (43). This interpretation is hypothesis-generating and based on temporal association rather than direct physiological measurement. In this context, the 24–48-hour delay should not be interpreted as immediate neurovascular crosstalk but rather as a delayed sensitization cascade following autonomic phase transition.

The stereotyped sequence of repetitive sneezing followed by prolonged watery rhinorrhea is consistent with an acute trigemino-parasympathetic reflex discharge, governed by the sphenopalatine ganglion and superior salivatory nucleus (11, 12, 45). Similar nasal autonomic responses have been described experimentally following stimulation of the sphenopalatine ganglion, resulting in vasodilation and plasma protein extravasation (45–47). In the present case, however, these findings should be interpreted as phenomenological parallels rather than evidence of identical underlying mechanisms (46).

Experimental data indicate that repetitive activation of parasympathetic efferents can sensitize meningeal nociceptors through the release of vasoactive intestinal peptide (VIP), nitric oxide, and CGRP (2, 3, 48). Extrapolating from these observations, a subsequent sympathetic rebound—via catecholaminergic drive and endothelin-1 signaling—may transiently disturb endothelial integrity and facilitate trigeminovascular activation (9, 31, 33, 36, 49).

Clinically, this autonomic phase transition was most evident after sleep, when cranial-autonomic symptoms were sometimes followed by temporary pain relief and, in some instances, delayed migraine onset (39, 40, 44). This “parasympathetic discharge” phase may represent a transient modulatory or defensive reflex influencing trigeminovascular input (3, 5, 46). Such biphasic autonomic patterns illustrate how parasympathetic and sympathetic influences may alternately predominate, shaping migraine expression in this individual (12, 49).

Overall, these observations align with broader conceptualizations of migraine as a disorder of dynamic homeostatic regulation rather than a purely paroxysmal pain syndrome (35, 37). Within this framework, autonomic imbalance, endothelial reactivity, and sensory hypersensitivity can be viewed as interacting dimensions of dysautonomia (38). While intriguing, this interpretation remains speculative and should be regarded as a clinical signal rather than mechanistic proof.

Such observations are consistent with the emerging view that migraine and trigeminal autonomic cephalalgias (TACs) may share overlapping autonomic–vascular pathways, while remaining clinically distinct entities (50).

### 2.6 Relationship to trigeminal autonomic cephalalgias (TACs)

TACs are characterized by prominent cranial-autonomic features—including tearing, nasal congestion, and eyelid edema—mediated by activation of the trigemino-parasympathetic reflex (45, 46, 50). In the present case, bilateral tearing, rhinorrhea, and repetitive sneezing similarly indicated cranial autonomic involvement.

Despite this overlap in autonomic manifestations, several clinical features distinguished the present pattern from TACs. The attacks were bilateral, lasted several hours, lacked strict orbital localization, and responded to triptans rather than indomethacin or oxygen—features inconsistent with cluster headache or paroxysmal hemicrania (46, 49, 50). Accordingly, the overall presentation remained most consistent with migraine without aura.

The presence of pronounced cranial-autonomic symptoms therefore suggests partial overlap at the level of autonomic pathways rather than diagnostic convergence. Migraine and TACs may share common reflex circuits while differing in attack structure, laterality, and systemic modulation (43, 44). In this context, autonomic activation should be interpreted as a dimension of clinical expression, not as evidence of a shared disease entity (9, 38).

### 2.7 Alternative interpretation considered

A purely hypertensive headache could theoretically explain symptom relief; however, hypertensive headaches are characteristically dull, diffuse, and temporally linked to acute pressure rises (47, 51). In this case, attacks long predated hypertension, were pulsatile and sensory-triggered, and resolved under therapy known to modulate endothelial and autonomic tone (9, 31). The remission therefore appears multifactorial—blood-pressure control likely contributed, while vascular–autonomic normalization may represent an additional contributing factor.

### 2.8 Future directions and research perspectives

Future studies could complement similar clinical observations with objective measures of endothelial and autonomic function, such as flow-mediated dilation (FMD), nitric-oxide metabolites (NOx), endothelin-1, CGRP, or heart-rate-variability (HRV) analysis (9, 31, 49). Such data would help clarify the physiological sequence linking endothelial reactivity and autonomic oscillation in migraine (7, 9).

At the genetic level, recent data suggest that endothelial nitric oxide synthase (eNOS) and CGRP receptor gene polymorphisms may influence individual response to vascular-targeted therapy (17, 32). This genetic variability could partly explain why antihypertensive and endothelial-modulating agents show heterogeneous efficacy across migraine populations, highlighting the need for phenotype- and genotype-based personalization in future studies (1, 17).

Digital phenotyping and continuous physiological monitoring may further refine autonomic–endothelial classification and guide personalized therapy (52).

### 2.9 Conceptual interpretation

The pharmacological and physiological interactions potentially relevant to the observed clinical course are summarized in Graphical Abstract. The figure integrates established endothelial and autonomic effects of combined antihypertensive therapy into a conceptual framework intended to contextualize the temporal association between treatment initiation and sustained migraine remission.

Importantly, Graphical Abstract should be interpreted as an integrative and hypothesis-generating model rather than a mechanistic pathway. It does not imply causal inference, mechanistic specificity, or exclusion of alternative explanations. Instead, it synthesizes previously discussed pharmacological and physiological observations in a manner consistent with the clinical findings of this single case (53).

A reflective commentary by the author is provided as Supplementary Material.

## 3 Limitations

Neuroimaging was limited to a normal head CT performed near the onset of symptoms, with no evidence of structural abnormalities. Magnetic resonance imaging (MRI) was not obtained, as the clinical presentation and long-term follow-up fulfilled ICHD-3 criteria for migraine without aura and no focal or systemic neurological signs emerged over more than ten years of observation.

Objective assessments of endothelial or autonomic function—such as circulating nitric oxide metabolites (NOx), endothelin-1, CGRP levels, heart-rate variability, or baroreflex sensitivity—were not performed, as these investigations are not routinely available in outpatient clinical practice. As a result, the proposed endothelial–autonomic interpretation is based on indirect clinical observations rather than direct physiological measurements. Therefore, mechanistic interpretations in this clinical case remain hypothesis-generating and should not be interpreted as physiological proof.

This manuscript reports a single clinical case, and its observations therefore cannot be generalized to the broader migraine population or considered evidence of a definitive pathophysiological mechanism. The proposed conceptual model should be regarded as hypothesis-generating, intended to illustrate a possible clinical pattern rather than to establish causal inference.

Nevertheless, carefully documented single-patient observations have historically contributed to advances in migraine research by highlighting phenotypic patterns that may be obscured in large heterogeneous cohorts. In the present case, the detailed temporal chronology and sustained remission under combined antihypertensive therapy provide a clinical signal that may help generate testable hypotheses for future studies employing objective endothelial and autonomic biomarkers in larger patient populations.

## 4 Conclusion

This case fulfilled all diagnostic features of migraine without aura and demonstrated complete, sustained remission following combined antihypertensive therapy.

While causal inference cannot be drawn from a single observation, the temporal course and accompanying vasomotor–autonomic manifestations are consistent with a pattern that may involve endothelial and autonomic modulation, alongside blood-pressure control. The findings illustrate how vascular and autonomic mechanisms may intersect in migraine pathophysiology and overlap partially with trigeminal autonomic cephalalgias, yet remain clinically distinct.

Recognition of such endothelium- and autonomic-linked clinical patterns could refine differential diagnosis and open research into vascular-targeted strategies for refractory migraine.

Future prospective work employing endothelial biomarkers (flow-mediated dilation, circulating NOx, endothelin-1) and heart-rate-variability metrics may further delineate this reversible spectrum.

## Data Availability

The datasets presented in this article are not readily available because of ethical and privacy restrictions. Requests to access the datasets should be directed to the corresponding author.
